# Molecular identification of *Lodoicea maldivica *(*coco de mer*) seeds

**DOI:** 10.1186/1749-8546-6-34

**Published:** 2011-09-30

**Authors:** Chun-yin Mak, Chuen-shing Mok

**Affiliations:** 1 Government Laboratory Hong Kong, 7/F Ho Man Tin Government Offices, 88 Chung Hau Street, Kowloon, Hong Kong SAR, China

## Abstract

**Background:**

The edible endosperm of *Lodoicea maldivica *with the common name of *coco de mer *is used in Chinese medicine for treating cough. Native to Seychelles, *Lodoicea maldivica *seeds have commanded high prices for centuries due to its scarcity. This study aims to develop a molecular identification method for the authentication of *Lodoicea maldivica *seeds.

**Methods:**

DNA was extracted from the sample. Two polymerase chain reaction (PCR) systems were developed to amplify a region of the chloroplast DNA and the nuclear phosphoribulokinase (PRK) region specific to *Lodoicea maldivica *respectively. DNA sequence of a sample was determined and compared with that of the *Lodoicea maldivica *reference material.

**Results:**

The PRK gene of *Lodoicea maldivica *was successfully amplified and sequenced for identification.

**Conclusion:**

A new molecular method for the identification of *Lodoicea maldivica *seeds in fresh, frozen or dried forms was developed.

## Background

*Lodoicea maldivica *(*coco de mer*), the sole member of the genus *Lodoicea*, is a fan-leaved palm native to Seychelles, bearing the largest and heaviest seed in the plant kingdom (weighing up to 30 kg). The seed is enclosed in a hard shell resembling a pair of coconuts joined in the middle. The seed is sometimes also referred to as the sea coconut, bum seed, double coconut, coco fesse or Seychelles nut. *Lodoicea maldivica *palms take between 25-50 years to reach maturity and bear fruit. The fruit may take one to three years to germinate [1]. Only two natural populations of *Lodoicea maldivica *remain [1]. Individual plants are also cultivated in various botanical gardens around the world. The harvest of seeds has virtually stopped all natural regeneration of the plant. The populations are also threatened by fire and encroachment by invasive plants. In March 2010, *Lodoicea maldivica *was added to the appendices of the Convention on International Trade in Endangered Species of Wild Fauna and Flora (CITES) in its 15^th ^meeting of the Conference of the Parties [2]. Trade of *Lodoicea maldivica *is also governed by the Protection of Endangered Species of Animals and Plants Ordinance in Hong Kong (Cap. 586). According to the ordinance, unless exempted, the import, introduction from the sea, export, re-export or possession of *Lodoicea maldivica *requires a licence issued by the Hong Kong government. The edible endosperm (*ie *fruit) of the plant has long been used in Chinese medicine for treating cough and is used mainly in soup in Hong Kong, China [3]. As fake products are often found in the markets, an identification method is needed.

This study aims to develop a new method for the identification of *Lodoicea maldivica *with DNA sequencing analysis. DNA was extracted from the edible endosperm sample. A polymerase chain reaction (PCR) system was designed to amplify a region of the chloroplast DNA to validate the quality of the extracted DNA [4,5]. A number of genes or spacer regions may be considered to identify unknown samples, for example, the internal transcribed spacer 1 [6], internal transcribed spacer 2 [6], nuclear ribosomal RNA genes 18S unit [6], large subunit of ribulose 1,5-bisphosphate carboxylase/oxygenase (*rbcL*) [7], intergenic region between the *β *subunit of ATP synthase (*atpB*) and *rbcL *[8] and the chloroplast trnL-trnF intergenic spacer region [9]. In this study, a PCR system targeting the nuclear phosphoribulokinase (PRK) region was designed. This region has been employed in many palm phylogenetics and shown ample characters to resolve relations at species level [10-13]. After DNA sequence analysis, the identity of the unknown was evaluated by pair-wise matching of its sequence with that of the certified *Lodoicea maldivica *reference species.

## Methods

### Materials

A *Lodoicea maldivica *seed certified by the Ministry of Environment, Natural Resources and Transport of the Republic of Seychelles was used as reference. The official certificate supports the authenticity of the species. The fresh reference seed is, thick, relatively hard and brown in colour with white flesh inside and about 10 kg in weight. The seed was assigned a voucher number of TD/TD/CRM/10001. The seed was kept frozen before analysis. Seven sliced commercial products with description of sea coconut or *coco de mer *(namely Sample A to Sample G) and one coconut sample (sample H) were purchased from the local market. Sample details are listed in Table 1

**Table 1:** Sample information and the results of their identities found

Sample code	Sample description (form)	Target region	Percentage identity	Identity found
Sample A	African seacoconut (dried)	PRK	99% [GenBank: (AF453357)]	*Lodoicea maldivica*
Sample H	Coconut (fresh)	PRK	100% [GenBank: (HQ265608)]	*Cocos nucifera*

For the samples in the form of pre-packaged product mixed with other materials, the suspected coconut portion was isolated from the background matrix before homogenisation. For fresh/frozen samples, the sample was homogenised by a blender, and about 1-5g of the sample was transferred into a flat bottom glass container. The sample was freeze-dried. The dried sample was ground into fine powder in liquid nitrogen. For dried samples, the sample was simply ground into fine powder in liquid nitrogen.

### DNA extraction

DNA extraction was performed with the CTAB method [14]. A total of 1.0 mL of pre-warmed (65°C) CTAB extraction buffer (2% w/v hexadecyl trimethylammonium bromide (CTAB), 0.1 M Tris, 1.4 M NaCl, 20 mM EDTA) was added to 100 mg of the test sample in a 2.0 mL microcentrifuge tube. Alpha-amylase solution (10 μL-20 μL), 20 μL of proteinase K solution (20 mg/mL), 100 μL of 10% w/v polyvinylpolypyrrolidone (PVP) and 5 μL of RNase A (100 mg/mL) were added. The mixture was incubated at 65°C overnight with constant agitation. After centrifugation (Eppendorf, Germany) for ten minutes at 16,000× g at room temperature, the supernatant was transferred to a new 2.0 mL tube and an equal amount of chloroform was added. The sample was shaken vigorously for 30 seconds before centrifugation (Eppendorf, Germany) for ten minutes at 16,000× g at room temperature. The upper layer was transferred to a new 2.0 mL tube and extracted with an equal amount of chloroform. The sample was centrifuged 16,000× g (Eppendorf, Germany) for five minutes. The upper layer was transferred to a new 2.0 mL tube and two volumes of CTAB precipitation solution (0.5% w/v CTAB, 0.04 M NaCl) were added. The sample was incubated at room temperature for 60 minutes without agitation. The supernatant was discarded after the sample was centrifuged (Eppendorf, Germany) for ten minutes at 16,000× g. The precipitate was dissolved in 350 μL of 1.2 M NaCl solution and was centrifuged (Eppendorf, Germany) for ten minutes at 16,000× g. The upper layer was transferred to a new 1.5 mL tube. Two volumes of cold absolute ethanol were added to precipitate the DNA. The sample was kept at -20°C for at least 20 minutes. The supernatant was discarded after centrifugation (Eppendorf, Germany) for ten minutes at 16,000× g at 4°C. A total of 500 μL of 70% cold ethanol solution was added and the sample was centrifuged (Eppendorf, Germany) for ten minutes at 16,000× g at 4°C. The DNA pellet was dried in a 37°C oven for several minutes and was then re-dissolved in 100 μL of DNase-free water.

### PCR for chloroplast DNA

The chloroplast DNA region (partial) was amplified with 50 ng DNA template, 1.0 μL of 5 mM dNTPs, 0.5 μL of 25 mM MgCl_2_, 2.5 μL of 10× PCR buffer plus MgCl_2 _(Applied Biosystems, USA), 0.5 μL of 10 μM specific primers (Table 2) and 1 U of AmpliTaq Gold polymerase (Applied Biosystems, USA). Water was added to make up to 25 μL. The DNA template was denatured at 95°C for five minutes and then 35 cycles of 30 seconds at 94°C, 30 seconds at 55°C, 45 seconds at 72°C and final extension at 72°C for five minutes. The amplification products were electrophoresed on a 1% agarose gel, stained with ethidium bromide and observed under UV illumination.

**Table 2:** Nucleotide sequences of specific primers used for the amplification of the chloroplast DNA and PRK gene

Name	Oligonucleotide DNA sequence (5'-3')	Amplicon size	Specificity
Plant 1 (forward)	CGA AAT CGG TAG ACG CTA CG	550 bp	Chloroplast DNA
Plant 2 (reverse)	GGG GAT AGA GGG ACT TGA AC		
prk717f (forward)	GTG ATA TGG AAG AAC GTG G	750 bp	PRK gene
prk969r (reverse)	ATT CCA GGG TAT GAG CAG C		

### PCR for PRK gene

The PRK gene was amplified with 50 ng DNA template, 1.25 μL of 5 mM dNTPs, 2.0 μL of 25 mM MgCl_2_, 2.5 μL of 10× PCR buffer without MgCl_2 _(Applied Biosystems, USA), 1.5 μL of 10 μM specific primers (Table 2), 1.25 μL of 40 ng/μL bovine serum albumin (BSA), 0.5 μL of dimethyl sulfoxide (DMSO) and 1 U of AmpliTaq Gold polymerase (Applied Biosystems, USA). Water was added to make up the final volume to 25 μL. The DNA template was denatured at 95°C for four minutes, and then 35 cycles of one minute at 94°C, 30 seconds at 54°C, one minute at 72°C and final extension at 72°C for seven minutes. The amplification products were electrophoresed on a 1% agarose gel, stained with ethidium bromide and observed under UV illumination.

### DNA sequencing

The PCR products of PRK gene were purified with DNA Clean & Concentrator™ -25 (Zymo Research, USA) according to the manufacturer's instructions. The BigDye^® ^Terminator v3.1 Cycle Sequencing Kit (Applied Biosystems, USA) was used for the cycle sequencing reaction. The PCR product was first denatured at 96°C for one minute and then 25 cycles of thermal cycling was performed as follows: 96°C for ten seconds, 50°C for five seconds, 60°C for four minutes. The BigDye^® ^XTerminator™ Purification Kit (Applied Biosystems, USA) was used to purify the extension products according to the manufacturer's instructions and the products were then run on the ABI PRISM^® ^3130XL Genetic Analyzer (Applied Biosystems, USA).

### Sequence Analysis

We aligned the sequence of the unknown sample in pair with that of the reference species using the bl2seq tool of the National Center for Biotechnology Information (NCBI) website [15]. Moreover, we performed multiple sequence alignment using the CLUSTAL2 tool of the European Bioinformatics Institute website [16].

## Results and Discussion

### DNA extraction

A study [17] reported that DNA from the solid endosperm of coconut was not a good starting material for molecular biology work because the matrix may hinder enzyme activity and because of the high lipid and polysaccharide content. The study suggested that DNA from young leaves of the plant should be used as the source of genomic DNA. Nevertheless, only the edible endosperm of *Lodoicea maldivica *is used in Chinese medicine and the commercial products thereof are made up of various forms of its edible endosperm. In order to develop a method for testing the authenticity of the claimed *Lodoicea maldivica *products, we think that PCR-ready DNA must be extracted from the endosperm of *Lodoicea maldivica*.

The chemical PVP is an important reagent in DNA extraction. Without the addition of PVP, the sample solution for the extracted DNA contained high levels of polysaccharide, as shown in the UV spectra. The contamination was greatly reduced by the addition of PVP during the initial incubation at 65°C in the extraction step. While the amount of DNA extracted from all samples in this study was very low (< 10 ng/μL), the quality of the extracted genomic DNA was sufficient for the PCR and sequencing.

### PCR-chloroplast DNA

Plant 1 and Plant 2 primers are targeted for the amplification of a region of plant chloroplast DNA and were used to verify the quality of the template DNA [2]. A single PCR product of about 550 bp was successfully amplified from all samples. The positive PCR results indicated that DNA of sufficient quantity and quality were successfully extracted and that the PCR was not inhibited by other components of the analytical sample.

### PCR PRK gene

The nuclear region of the PRK gene was amplified with the second PCR system. This region has been used successfully to resolve palm phylogenetic relations at the species level [10-13]. Positive results (*ie *a fluorescent DNA band of about 750 bp) from this PCR system would indicate that the sample contained the PRK gene whereas negative results would indicate that the sample was not from *Lodoicea maldivica*.

Single-band PCR products of about 750 bp were successfully amplified from *Lodoicea maldivica *reference material and two commercial products namely Sample A and Sample H. Other samples yielded either negative results or multiple PCR products in the amplification of PRK gene. *Lodoicea maldivica *yielded only one single PCR product in the analysis of PRK gene. The sample should be considered as not of *Lodoicea maldivica *in origin when no or more than one band was observed after agarose gel electrophoresis [4]. The identities of these unknown samples were found by using one of the markers [6-9] as mentioned in the background section.

### Sequence analysis

**Figure 1:**
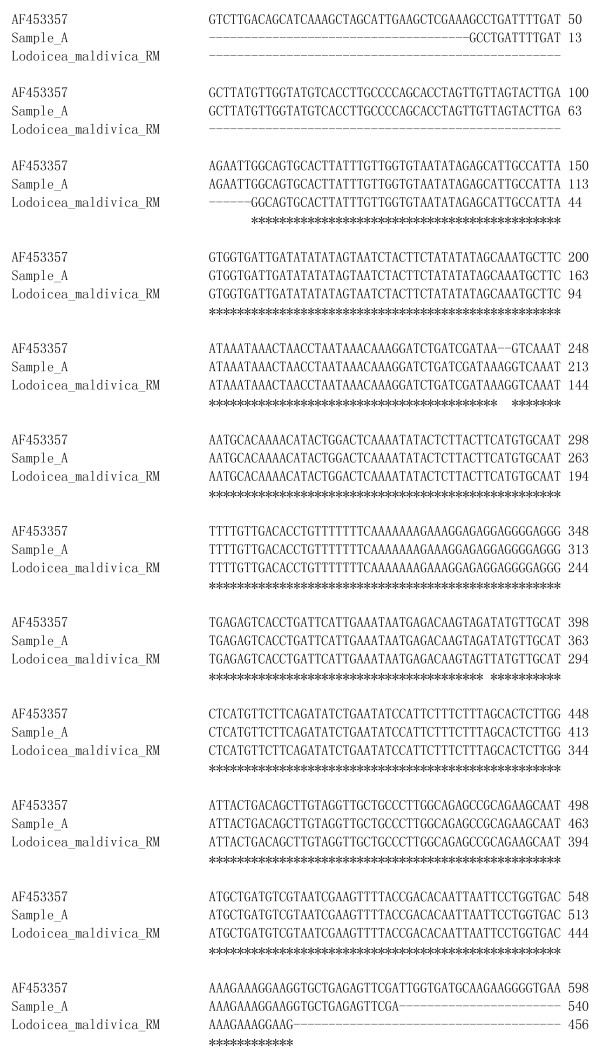
**Sequences alignment for *Lodoicea maldivica *reference material (RM) [GenBank: **JF820816], **Sample A and [GenBank: **AF453357].

**Figure 2:**
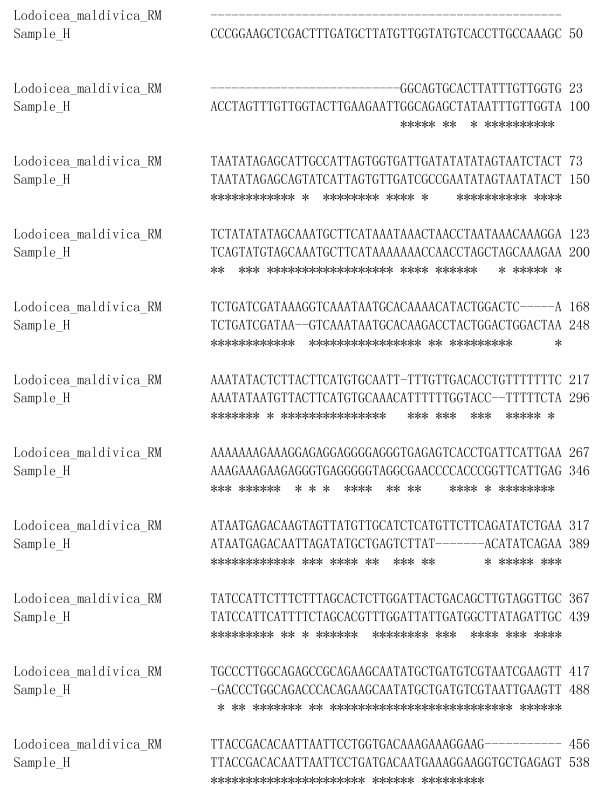
**Sequences alignment for *Lodoicea maldivica *reference material (RM) [GenBank: **JF820816] **and Sample H**.

**Figure 3:**
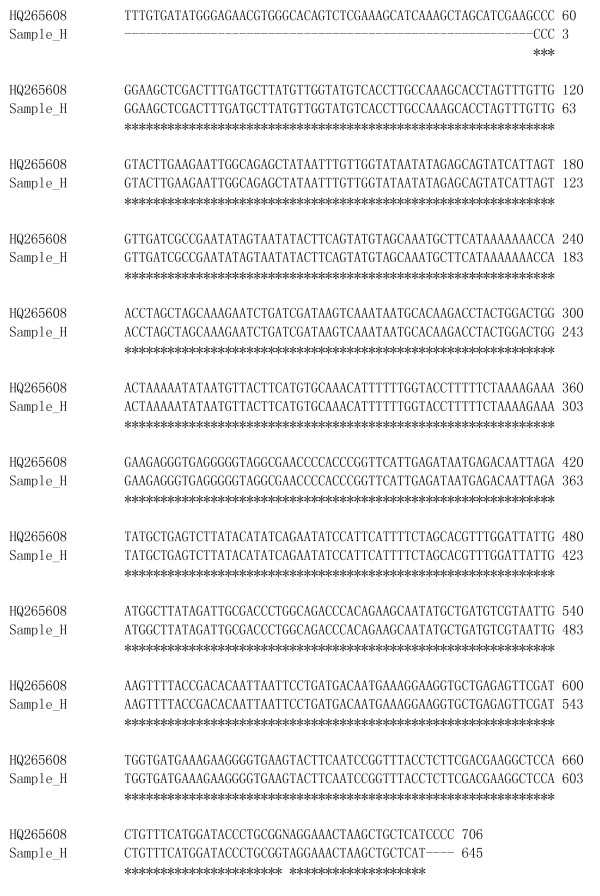
**Sequences alignment for Sample H and *Cocos nucifera *[GenBank: **HQ265608].

All the amplified PRK genes were successfully sequenced. The nucleotide sequence of the *Lodoicea maldivica *reference material was submitted to the GenBank [GenBank: JF820816]. We compared the nucleotide sequence in pair between the reference material and each of the unknown samples using the bl2seq tool [15]. For those sequences not matching the reference material, further search in the GenBank was performed. The PRK gene sequence of sample A was identical to that of the reference material (Figure 1). While the PRK gene sequence of sample H only matched that of the reference material with 80% homology (Figure 2), it matched the PRK sequence of species *Cocos nucifera *with 100% homology in GenBank database [GenBank: HQ265608] (Figure 3). Currently, there is only one single entry of the *Lodoicea maldivica *PRK gene sequence in NCBI GenBank [GenBank: AF453357]. The sequence was obtained from a *Lodoicea maldivica *voucher (voucher number: C.E. Lewis 98-020/BH) and reported by Lewis *et al*. in 2002 [12]. However, the exact source of the voucher was not reported. The BLAST [15] result of the *Lodoicea maldivica *reference material sequence obtained in this study showed a 99% homology to that of the [GenBank: AF453357] (Figure 1). The *Lodoicea maldivica *reference species used in this study was obtained from the country of origin of the species with official certificate. The present study provides an alternative source of nucleotide sequence data for GenBank for the identification of *Lodoicea maldivica*. The applicability and accuracy of the method have been demonstrated by the consistent results obtained from replicated analysis of the samples.

## Conclusion

A new molecular method for the identification of *Lodoicea maldivica *seeds in fresh, frozen or dried forms was developed.

## Abbreviations

CITES: Convention on International Trade in Endangered Species of Wild Fauna and Flora; CTAB: hexadecyl trimethylammonium bromide; DMSO: Dimethyl sulfoxide; EDTA: Ethylenediaminetetraacetic acid; NCBI: National Center for Biotechnology Information; PCR: polymerase chain reaction; PRK: phosphoribulokinase; PVP: polyvinylpolypyrrolidone.

## Competing interests

The authors declare that they have no competing interests.

## Authors' contributions

CYM conceived and designed the study, collected the samples, performed the laboratory work, analyzed and interpreted the data, and drafted the manuscript. CSM supervised the project and finalized the manuscript. Both authors read and approved the final version of the manuscript.

## Disclaimers

This article does not necessarily reflect the views of the Government of the HKSAR. The mentions of trade names or commercial products do not constitute any endorsement or recommendations.
